# Different Magnetization Levels of Magnetite–Chitosan Nanocomposites for Co (II) Adsorption from Natural Waters

**DOI:** 10.3390/nano16070393

**Published:** 2026-03-25

**Authors:** Sergej Šemčuk, Živilė Jurgelėnė, Vidas Pakštas, Danguolė Montvydienė, Audrius Drabavičius, Kęstutis Jokšas, Martynas Talaikis, Jonas Mažeika, Kęstutis Mažeika, Karina Kuzborskaja, Galina Lujanienė

**Affiliations:** 1 State Research Institute Center for Physical Sciences and Technology (FTMC), Savanorių ave. 231, 02300 Vilnius, Lithuania; vidas.pakstas@ftmc.lt (V.P.); audrius.drabavicius@ftmc.lt (A.D.); martynas.talaikis@ftmc.lt (M.T.); kestutis.mazeika@ftmc.lt (K.M.); karinakuzborskaja@gmail.com (K.K.); galina.lujaniene@ftmc.lt (G.L.); 2 State Scientific Research Institute Nature Research Centre (NRC), Akademijos St. 2, 08412 Vilnius, Lithuania; zivile.jurgelene@gamtc.lt (Ž.J.); danguole.montvydiene@gamtc.lt (D.M.); kestutis.joksas@gamtc.lt (K.J.); jonas.mazeika@gamtc.lt (J.M.)

**Keywords:** magnetic biopolymers, Fe_3_O_4_ loading, metal removal, cobalt, radionuclide removal, adsorption kinetics, adsorption isotherms, natural waters

## Abstract

Biopolymers such as chitosan are considered important candidates for water purification due to their non-toxicity, biodegradability, natural origin, biocompatibility, and potential for modification to provide additional capabilities, such as incorporating nanomaterials for magnetism to enable rapid separation or adding functional groups to enhance selectivity towards target adsorbates. This study investigated adsorption of Co (II), traced by Co-60 radionuclide, systematically evaluated in natural aquatic matrices selected according to water body type: seawater (Baltic Sea) and freshwater systems further distinguished as lentic (Lake Balsys) and lotic (Neris River) environments, using synthesized magnetite–chitosan nanocomposites (MCNs) with varying loadings of Fe_3_O_4_ (10–30 wt. %) nanoparticles providing different levels of magnetization. Comprehensive characterization (TEM, FTIR, AFM, XRD, and Mössbauer spectroscopy) confirmed successful integration of magnetite nanoparticles within the chitosan matrix and reproducible structural properties. An optimal magnetization of 11 emu/g was achieved at 20 wt. % Fe_3_O_4_, enabling rapid magnetic separation within <1 min without compromising sorption capacity. Adsorption isotherm models were applied to investigate the adsorption parameters, and sorption kinetics were studied, yielding a maximum adsorption capacity of 14.93 mg/g for MCN-10 in seawater and 11.95 mg/g for MCN-20 in freshwater with observed equilibrium within 120 min. These promising results indicate that the MCN is a suitable nanocomposite for the removal of Co (II) ions and the Co-60 radionuclide from aquatic media.

## 1. Introduction

Natural water resources are increasingly impacted by anthropogenic pollution. Industrial wastewater discharges have been demonstrated to introduce a wide range of contaminants into drinking water, including heavy metals, organic pollutants, and radionuclides (e.g., Zn, Ni, Cr, Hg, Cd, Cu; fertilizers, pesticides, pharmaceuticals; Eu, Co, Cs) [1]. The combined effects of climate change and urbanization have been demonstrated to enhance the dispersal of pollutants and diminish freshwater availability, resulting in elevated contaminant concentrations and an augmentation in the incidence of emerging pollutants within surface and groundwater systems [2]. Environmental monitoring studies indicate that cobalt is commonly present in natural waters at low concentrations. In surface and groundwater, cobalt concentrations are typically <1 μg/L in pristine environments and approximately 1–10 μg/L in populated areas, although significantly higher levels may occur near mining and industrial activities [3]. In heavily contaminated locations affected by mining or metallurgical operations, cobalt concentrations in surface waters have been reported to reach several thousand μg/L, demonstrating the potential for substantial environmental contamination in impacted regions.

Conventional wastewater treatment processes often prove ineffective in the removal of these contaminants due to their high stability, small molecular size, or the absence of selective removal technologies. Even small discharges of heavy metals in wastewater or trace amounts of radionuclides can irreversibly compromise environmental integrity [4,5] due to their high toxicity, non-biodegradability, increased solubility and mobility in water systems, and their ultimate threat to animal health and, consequently, human health through entry into food chains [1,6]. Radionuclides such as 60-Co are of particular concern because they present both chemical toxicity and radiological hazards. These substances have been shown to substitute for biologically essential metals, thereby disrupting enzymatic processes in aquatic organisms. Additionally, their long half-life and mobility have been demonstrated to increase the risk of chronic low-dose exposure, which can lead to carcinogenic and mutagenic effects [7,8].

Among established water treatment technologies, adsorption remains one of the most efficient and technically straightforward approaches due to its strong ability to remove diverse contaminants and its operational flexibility. The adsorption on nanoporous and micro- or nanoscale materials, including activated carbon, zeolites, functionalized silica, polymers, and clay-based sorbents, has been shown to enable effective removal through surface interactions such as electrostatic attraction, complexation, and ion exchange. Adsorption can be readily coupled with complementary processes, such as advanced oxidation or membrane filtration, to form hybrid systems with enhanced performance and reduced treatment costs. Recent advancements in the field of sorbent engineering, particularly the development of hierarchically structured materials, stimuli-responsive polymers, and three-dimensional nanostructured frameworks, have further enhanced mass transfer, selectivity, and adsorption capacity [9,10].

Biopolymers such as chitosan have emerged as attractive materials for water treatment due to their non-toxicity, biodegradability, natural abundance, and biocompatibility. Chitosan can be readily modified to improve adsorption capacity, mechanical stability, and selectivity toward specific contaminants. The functionalization of amino acids, thiol, carboxyl, or polyphenolic groups enables targeted binding of metal ions and organic pollutants under varying solution conditions [11,12,13,14]. The incorporation of magnetic nanocomponents, such as iron oxides, into chitosan-based sorbents has been shown to enhance their performance. In addition to enabling magnetic properties, magnetite (Fe_3_O_4_) nanoparticles may contribute directly to the adsorption mechanism by providing additional active surface sites [15]. Surface hydroxyl groups on magnetite can interact with metal ions through electrostatic attraction or surface complexation mechanisms. Furthermore, the presence of Fe_3_O_4_ nanoparticles can increase the effective surface area of the composite and improve the distribution of adsorption sites within the chitosan matrix. As a result, magnetic chitosan composites combine the chelation ability of amino groups in chitosan with additional adsorption interactions occurring on magnetite surfaces. This enhancement is achieved by facilitating rapid magnetic separation and efficient recovery from water, thereby reducing the need for energy-intensive filtration or centrifugation processes [1]. These magnetic chitosan composites demonstrate excellent reusability and recyclability, positioning them as promising candidates for scalable water treatment and advanced applications, including selective radionuclide capture and responsive separation systems [16,17].

In the present study, magnetite–chitosan nanocomposites with systematically varied Fe_3_O_4_ loadings (10–30 wt. %) are investigated for the removal of Co (II), traced with ^60^Co, from natural water matrices. By isolating magnetization as a design parameter, this study aims to elucidate how Fe_3_O_4_ loading governs the balance between magnetic separability and chitosan-based adsorption performance in complex aqueous matrices. This aspect has not been sufficiently explored in the current literature [18,19]. In accordance with the findings of recent assessments of water quality management and treatment scale-up strategies [20], in our case, the use of environmentally relevant water matrices (sea, river, lake) allows for an assessment of sorbent behavior under realistic conditions and may inform the development of biopolymer-based approaches for heavy metal and radionuclide removal. Comprehensive structural and magnetic characterization was employed to elucidate structure–property relationships relevant to adsorption performance. Subsequent analysis of the adsorption behavior was conducted through the implementation of equilibrium and kinetic modeling techniques. This approach enabled the quantitative benchmarking and mechanistic interpretation of the process under conditions that are relevant to the environment [21,22].

The obtained results demonstrate the potential of magnetite–chitosan nanocomposites for the efficient removal of heavy metals and radionuclides, provide easy collection using a magnetic field within 1 min, and suggest applicability to other emerging contaminants relevant to environmental and human health protection.

## 2. Materials and Methods

### 2.1. Synthesis of Adsorbents

The preparation of magnetic chitosan involved two steps: synthesis of Fe_3_O_4_ nanoparticles and modification of chitosan using a crosslinking method. This sequence was chosen to avoid potential variations in magnetite nanoparticle size and composition that can affect the magnetic properties and to ensure precise control of the load (10%, 20%, and 30%) by using synthesized Fe_3_O_4_ nanoparticles from a single synthesis. Another important parameter is the homogeneity of distribution, which was achieved through sonification and high-speed stirring during the modification of chitosan.

#### 2.1.1. Magnetite Synthesis

Magnetite (Fe_3_O_4_) was synthesized by mixing prepared solutions of iron (II) chloride tetrahydrate (CAS No. 134-10-9) and iron (III) chloride (CAS No. 7705-08-0) (Sigma Aldrich, Darmstadt, Germany), with 100 mL of each prepared under argon atmosphere, as described in [23].

$$2{FeCl}_{3}+{FeCl}_{2}+8{NH}_{4}OH\to {Fe}_{3}O_{4}+8{NH}_{4}Cl+4H_{2}O$$

Based on our previous investigations [24], the final magnetite composition is highly sensitive to the Fe^2+^:Fe^3+^ molar ratio, which is 1:2 (according to the reaction equation). To maintain this ratio and prevent the unwanted oxidation of Fe^2+^, the synthesis was performed under a strictly inert atmosphere using argon. Throughout the growth process, the solution was subjected to simultaneous mechanical stirring and ultrasonication. This approach was employed to provide high shear forces, preventing the formation of large polycrystalline aggregates and ensuring a more uniform particle size. The pH was adjusted via dropwise addition of ammonium hydroxide (CAS No. 1336-21-6, Honeywell Fluka, Seelze, Germany) to avoid agglomeration centers. This slow adjustment, combined with further use of a thermostatic bath for precise isothermal control where the temperature was raised to 75 °C, allowed us to decouple the nucleation burst from the growth phase. After 2 h of intensive mixing, the black magnetite nanoparticles were separated using a neodymium magnet, washed with ethanol and Milli-Q (Type I) water to neutral pH, and dried at 50 °C.

#### 2.1.2. Modification of Chitosan

Chitosan powder (100,000–300,000 m.w., Sigma Aldrich, Germany) was used for modification with magnetite nanoparticles in ratios of 7:3, 4:1, and 9:1, resulting in 30, 20, and 10 wt. % of Fe_3_O_4_ load in each magnetite–chitosan nanocomposite (MCN-30, MCN-20, and MCN-10), respectively. A 2% acetic acid solution was used to dissolve chitosan (1.5 g/100 mL *m*/*v*). Magnetite was dispersed in Milli-Q ultra-pure (type I) water by sonication. The required amounts of the prepared solutions were mixed in a flask to achieve the correct ratio and stirred for 2 h under an argon atmosphere. Subsequently, a 25% glutaraldehyde solution, as a crosslinking agent in a ratio of 1:100 to chitosan, was added dropwise to the mixture, followed by 2 h of mixing at 50 °C, with a gradual increase in pH to 9 using sodium hydroxide (NaOH 0.5 M) solution [25,26,27]. The resulting MCNs were collected using a neodymium magnet. Acetonitrile was used to improve separation of the formed MCN flakes, which were then washed with ethanol and Milli-Q water until neutral pH was reached. After freeze-drying and homogenization for improved dosing, the MCNs were characterized and used in the sorption study.

### 2.2. Characterization of Synthesized Materials

The structural, surface and chemical-physical properties of synthesized magnetite and magnetite–chitosan nanocomposites were studied using various techniques. The surface morphology was imaged using transmission electron microscopy (TEM) on a Tecnai G2 F20 X-TWIN with a Schottky-type field emission electron source (FEI, Eindhoven, Netherlands). The XRD patterns were recorded with X-ray diffractometer Smart Lab equipped with an X-ray tube with a 9-kW rotating Cu anode (Rigaku, Tokyo, Japan). XRD measurements were performed using Bragg–Brentano geometry with a graphite monochromator on the diffracted beam, in step scan mode with a step size of 0.02° (2θ scale) and a counting time of 1 s per step. Measurements were taken in the 2θ range from 10° to 75°. Phase identification was performed using the PDXL2 software package (Rigaku) and the ICDD powder diffraction database PDF4+ (2023 release). Fourier transform infrared (FTIR) spectra were obtained using an Alpha spectrometer (Bruker, Inc., Ettlingen, Germany) equipped with a room temperature RT-DLATGS detector and attenuated total reflectance (ATR) accessory, Platinum ATR Diamond (Bruker Inc., Ettlingen, Germany). Mössbauer spectra were measured using a Mössbauer spectrometer (Wissenshaftliche Elektronik GmbH, Starnberg, Germany) with a ^57^Co (Rh) source. Magnetization curves were recorded using a magnetometer consisting of an SR510 lock-in amplifier (Stanford Research Systems, Sunnyvale, CA, USA), an FH-54 Gauss/Tesla meter (Magnet Physics, Cologne, Germany), and a laboratory magnet supplied by an SM 330-AR-22 power source (Delta Electronica, Zierikzee, The Netherlands).

### 2.3. Point of Zero Charge

The point of zero charge (*pH_pzc_*) for MCN-10, MCN-20, and MCN-30 was determined using the solid addition method [26]. The initial pH (*pH_i_*) of a 0.1 M NaCl solution was adjusted to 2, 3, 4, 5, 6, 7, 8, 9, 10, and 11 using 0.1 M NaOH and 0.1 M HCl solutions. All pH during all sets of measurements were recorded with a WTW inoLab Multi-Level 1 pH meter and a WTW SenTix 81 electrode (Xylem Analytics, Weilheim, Germany). The same amount of each MCN was then added to the samples, which were shaken continuously for 24 h at 20 °C. After separation of MCN-10, MCN-20, and MCN-30 using a magnetic field, the pH of each sample was measured (*pH_eq_*). Several measurements were conducted for each sample, and a change of pH (Δ*pH*) was calculated using Equation (1). Afterward, the averages of measurements were plotted for analysis.

(1) $$∆pH={pH}_{i}-{pH}_{eq}$$

### 2.4. Natural Water Samples Used for Adsorption Studies

Natural waters were used in adsorption studies to ensure compliance with real conditions of seawater and freshwater in stationary and flowing modes. Respectively, Baltic Sea, Lake Balsys and Neris River water samples were collected over one week in the middle of April 2025.

Baltic Sea (BS) water samples were collected near the Būtingė terminal (56.053405° N, 21.058396° E). The typical salinity of BS water in this area ranges from 5.0‰ to 7.1‰, with an average annual value of approximately 6.2‰. The pH ranges from 6.76 to 7.74, with an average annual value of 7.29 [28].

Lake Balsys (LB) is a popular local area for relaxation during the warm season in Vilnius (54.787206° N, 25.334148° E). The typical pH of LB water is about 8; more detailed water characterization is available in our previous article [29].

Neris River (NR) water samples were collected near Buivydžiai (54.838940° N, 25.74192° E), far away from strongly populated territories. Yearly Neris River water composition monitoring data can be found in [30].

Prior to the adsorption experiments, the water samples were filtered through a 1 µm glass fiber filter to remove particulate matter, including sand and microorganisms, thereby minimizing changes in water composition. The Co (II) concentrations in the samples were below the detection limit (<0.1 µg/L), thereby excluding any potential influence of cobalt ions from the tested waters on the results. The pH of the water samples was measured using a WTW inoLab pH meter with a WTW SenTix 81 electrode (Xylem Analytics, Germany). The registered pH values at 10 °C in used water samples for experiments were as follows: Baltic Sea 7.58 (BS), Lake Balsys 7.77 (LB), and Neris River 7.85 (NR).

### 2.5. Batch Adsorption Experiments

Batch sorption experiments were conducted on the BS, LB, and NR water samples with initial Co (II) ion concentrations ranging from 1.25 to 20 mg/L, achieved by dissolving the required amount of CoCl_2_·6H_2_O. The selected concentrations facilitated the assessment of the sorption properties of the sorbents under study; in the event of an accident, similar or higher concentrations are likely. For the sorption kinetics study, an initial Co^2+^ concentration of 1.25 mg/L was used. All samples were spiked with the same amount of Co-60 radionuclide; subsequent changes in ^60^Co concentration reflected changes in Co^2+^ concentration. The experiments were carried out at 15 °C with gentle shaking throughout the exposure periods. After the required contact time, ranging from 5 to 1440 min for the kinetics study and 24 h for the initial concentration dependence, the MCNs were separated using a neodymium magnet, and the solution was transferred to a designated sample measurement box for the determination of ^60^Co concentration changes using a gamma spectrometer (CANBERRA PACKARD, Schwadorf, Austria) with a germanium detector (5 kV/1.4 MeV, efficiency 42%). The measurement results were used to evaluate the removal efficiency and sorption capacity of MCN-10, MCN-20, and MCN-30 nanocomposites for the sorption of Co (II) ions in BJ, LB, and NR water samples using Equations (2) and (3) for calculations:

(2) $$RE=\frac{C_{0}-C_{e}}{C_{0}}\times 100\%$$

(3) $$Q_{e}=\frac{\left(C_{0}-C_{e}\right)\cdot V}{m}$$

where *RE* (%) is the removal efficiency of MCN’s, *C*_0_ and *C_e_* (mg/L) represents the initial and final concentrations of Co (II) in the solution, *Q_e_* (mg/g) is the adsorption capacity, *V* (L) is the volume of sample, and *m* (g) is the mass of used MCNs.

### 2.6. Adsorption Isotherms and Kinetic Models

The results of the MCN sorption capacity for Co (II) obtained from the sorption experiment were analyzed using linear forms of five adsorption isotherms: Freundlich, Langmuir, Temkin and Harkins–Jura. The quality of fit was assessed with the correlation coefficient (*R*^2^).

#### 2.6.1. Freundlich Isotherm

The Freundlich adsorption isotherm model [31] describes the adsorption of adsorbate onto a heterogeneous surface of adsorbent (Equation (4)). Parameter *K_F_* indicates the adsorption capacity, while *n* shows the adsorption intensity. There are two methods of interpretation for the calculated *n* value for favorable adsorption: either when it fulfils the condition 0 < 1/*n* < 1 [16] or when *n* > 1 [32].

(4) $$\mathrm{Log}\;Q_{e}=\log K_{F}+\frac{1}{n}\log C_{e}$$

#### 2.6.2. Langmuir Isotherm

The Langmuir adsorption isotherm model [33] is based on the assumption that the adsorption of adsorbate occurring on the surface of adsorbent is monolayered [32]. The maximum adsorption capacity for complete monolayer coverage *Q_max_* (mg/g) and the *K_L_* (L/mg) adsorption constant related to the energy of adsorption are the parameters from the isotherm (Equation (5)). The separation factor *R_L_* (L/mg) (Equation (6)) can be calculated to indicate the adsorption type: unfavorable (*R_L_* > 1), linear (*R_L_* = 1), favorable (0 < *R_L_* < 1), or irreversible (*R_L_* = 0) [34].

(5) $$\frac{C_{e}}{Q_{e}}=\frac{1}{Q_{max}}C_{e}+\frac{1}{K_{L}Q_{max}}$$

(6) $$R_{L}=\frac{1}{1+{K_{L}C}_{0}}$$

#### 2.6.3. Temkin Isotherm

The Temkin adsorption isotherm model [35] includes the heat of adsorption reduction in various adsorbent layers probably caused by the interaction between adsorbents and adsorbates. Its linear form is shown in Equation (7) with the following parameters: *K_T_* (L/g) is the maximum equilibrium binding constant and *B* (J/mol) is related to the adsorption heat constant that describes the heterogeneity of the surface of adsorbent [12,36].

(7) $$Q_{e}=\mathrm{B ln}K_{T}+B\ln C_{e}$$

#### 2.6.4. Harkins–Jura Isotherm

The Harkins–Jura adsorption isotherm model [37] is based on the concept of multilayer adsorption on adsorbents with heterogeneous pores [38,39]. For aqueous phase adsorption, the linear form shown in Equation (8) is commonly used, where *A_HJ_* and *B_HJ_* are Harkins–Jura isotherm model constants, which suggests that a plot of 1/*Q_e_*^2^ against ln(c) will yield a straight line [40].

(8) $$\frac{1}{Q_{e}^{2}}=\frac{B_{HJ}}{A_{HJ}}–\frac{1}{A_{HJ}}l\mathrm{og}C_{e}$$

#### 2.6.5. Adsorption Kinetic Models

The obtained data during the kinetic experiments was calculated by Equation (9), where *Q_t_*, (mg/g) is the amount of adsorbate adsorbed at time *t*, *t* (min) is the contact time of adsorbate and adsorbent, *C*_0_ represents the initial and *C_t_* (mg/L) registered concentrations of Co (II) in the solution at time t, *V* (L) is the volume of sample, and *m* (g) is the mass of used MCN.

(9) $$Q_{t}=\frac{\left(C_{0}-C_{t}\right)\cdot V}{m}$$

The obtained results of dependence on contact time (*t* vs. *Q_t_*) were plotted and analyzed using the OriginPro 2025 (Northampton, MA, USA) program by the non-linear fit of pseudo-first-order (PFO), pseudo-second-order (PSO) and Elovich kinetic model’s Equations (10)–(12) [41], respectively:

(10) $$Q_{t}=Q_{e}(1-e^{-k_{1}t})$$

(11) $$Q_{t}=\frac{Q_{e}^{2}k_{2}t}{1+Q_{e}k_{2}t}$$

(12) $$Q_{t}=\frac{1}{\beta }ln(\alpha \beta t+1)$$

where *Q_e_* (mg/g) is the adsorption capacity at equilibrium; *t* (min) is the contact time of adsorbate and adsorbent; *k*_1_ (1/min) is the pseudo-first-order rate constant; *k*_2_ (g/mg·min) is the pseudo-second-order rate constant; *α* (g/mg·min) is the Elovich rate constant indicating the initial rate; and *β* (g/mg) is the Elovich rate constant, indicating the desorption rate to surface coverage and activation energy.

The quality of fit was assessed with correlation coefficients (*R*^2^) and the chi-square test (*χ*^2^).

## 3. Results and Discussion

### 3.1. Characterization

#### 3.1.1. Transmission Electron Microscopy (TEM)

**Figure 1:**
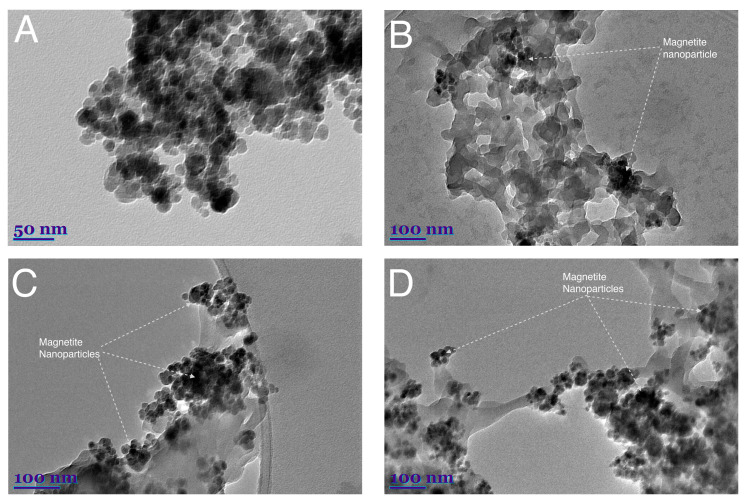
TEM images of synthesized magnetite (**A**) and magnetite–chitosan nanocomposites MCN-10 (**B**), MCN-20 (**C**), MCN-30 (**D**).

TEM images of synthesized magnetite nanoparticles are shown in Figure 1A at a 50 nm scale and magnetite–chitosan nanocomposites are shown at a 100 nm scale (B, C, D). The size of the synthesized Fe_3_O_4_ nanoparticles ranged from 15 nm to 25 nm, with an average of 18 nm (Figure 1A). Similar particle sizes were observed in the images of synthesized MCN-10 (B), MCN-20 (C), and MCN-30 (D). The magnetic nanoparticles typically attached to the edge of the biopolymer and did not alter its morphology. They also did not damage or interact with other active groups that were inaccessible due to distance, and the polymer did not collapse or intertwine. Thus, these “free” amino and hydroxy groups could participate in adsorption processes or be further modified. Moreover, images C and D clearly show that magnetite nanoparticles formed significant agglomerates compared to B, providing improved magnetic properties for the synthesized MCN-20 and MCN-30. The resulting MCN nanocomposites consisted of micron-sized particles decorated with magnetite nanoparticles. In addition, the quantitive size distribution was observed using an SEM image of MCN-30 (Supplementary Figure S1a). The size of the nanocomposite varied up to 25 µm, with average size of 3.32 µm (Supplementary Figure S1b).

#### 3.1.2. X-Ray Diffraction (XRD)

**Figure 2:**
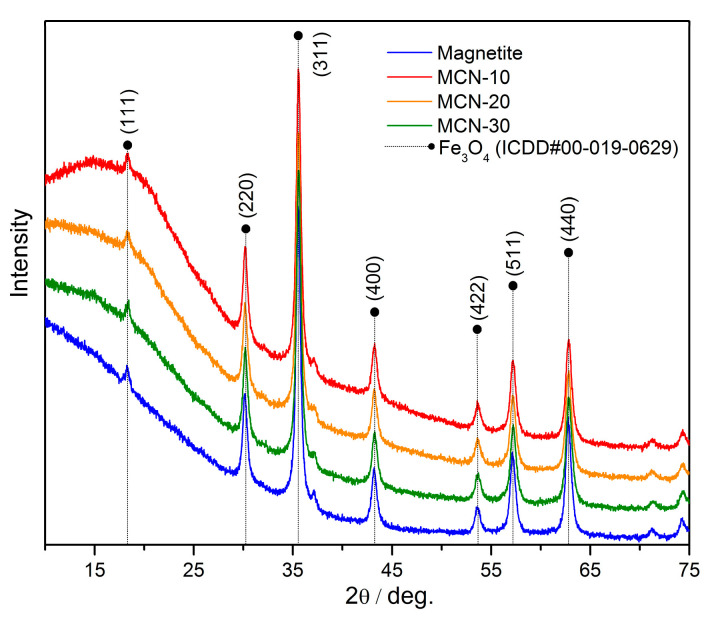
XRD patterns of synthesized magnetite, MCN-10, MCN-20, and MCN-30, with typical Fe_3_O_4_ diffraction peaks.

The XRD patterns recorded for magnetite, MCN-10, MCN-20, and MCN-30 are presented in Figure 2. Characteristic diffraction peaks are observed at 2θ = 18.33°, 30.15°, 35.52°, 43.17°, 53.56°, 57.09°, and 62.69°. Based on the PDF-4+ database, the characteristic diffraction peaks of Fe_3_O_4_ (ICDD card no. #00-019-0629) are expected at the (111), (220), (311), (400), (422), (511), and (440) crystal planes, showing good agreement with the obtained XRD patterns [27,42]. The reduced intensity of the detected peaks is attributed to the presence of chitosan in the composition. The crystallite sizes of Fe_3_O_4_ nanoparticles alone and in synthesized MCNs samples were determined from the broadening of XRD peaks using the graphical Halder–Wagner method implemented in PDXL software. The results were as follows: magnetite 17.2 ± 1.8 nm, MCN-10 14.8 ± 1.4 nm, MCN-20 12.7 ± 1.7 and MCN-30 13.3 ± 1.8 nm. The results indicate that the crystal structure of magnetite remained unchanged during the synthesis of MCNs. These findings indicate a slight size decrease in magnetite nanoparticles after immobilization onto chitosan and that the crystal structure of magnetite remained unchanged during the synthesis of MCNs, providing preservation of magnetic properties in all cases.

#### 3.1.3. Fourier Transform Infrared Spectroscopy (FTIR)

**Figure 3:**
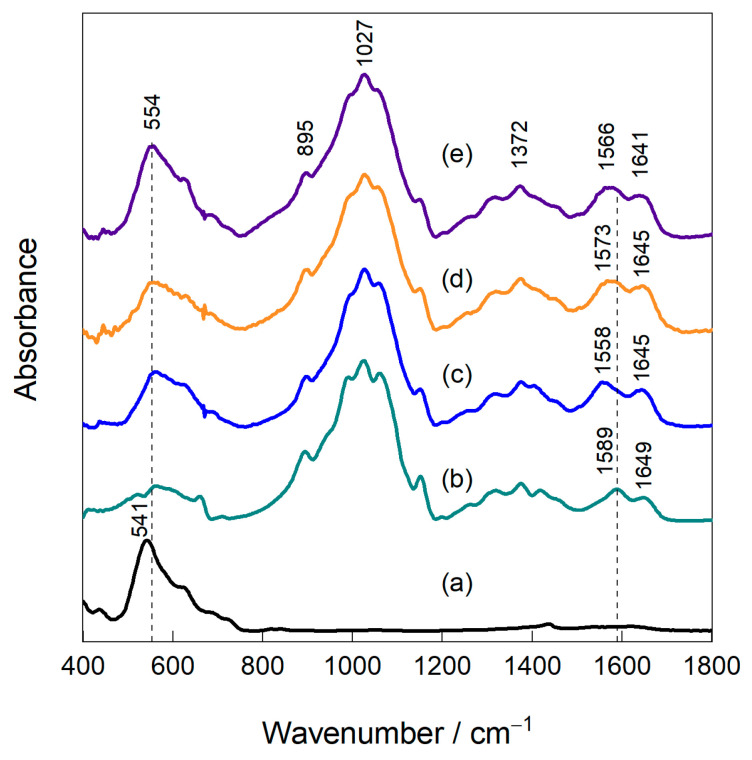
FTIR spectra of magnetite (a), chitosan (b), MCN-10 (c), MCN-20 (d), and MCN-30 (e).

Figure 3 shows the FTIR spectra of magnetite (a), chitosan (b) and magnetite–chitosan nanocomposites MCN-10 (c), MCN-20 (d), and MCN-30 (e), collected at a spectral resolution of 4 cm^−1^ from 50 scans and recorded in the range of 400–1800 cm^−1^. The characteristic absorption band observed at approximately 551 cm^−1^ corresponds to the Fe–O stretching vibrations of Fe_3_O_4_ (Figure 3 (a)) and is consistent with values reported in the literature for magnetite [43,44]. The spectrum of chitosan (Figure 3 (b)) exhibits a characteristic region at 1020–1050 cm^−1^, with a peak at approximately 1027 cm^−1^, corresponding to C–O–C and C–O stretching vibrations associated with the polysaccharide structure. The absorption band observed approximately at 1589 cm^−1^ is assigned to N–H bending vibrations of the amino groups, while the band at approximately 1649 cm^−1^ is attributed to C=O stretching of residual acetylated units. The spectra of all magnetite–chitosan nanocomposites (MCN-10, -20, -30; Figure 3 (c)–(e)) retained the characteristic absorption bands of both magnetite and chitosan, indicating successful formation of the nanocomposite. All MCN samples exhibited a prominent adsorption band at approximately 554 cm^−1^, which shifts significantly to 541 cm^−1^, attributed to Fe–O, and with intensity increasing as the magnetite content increases. The slight shifts and intensity changes in the 1020–1050 cm^−1^ region, with a peak at about 1027 cm^−1^, associated with C–O–C and C–O vibrations, compared to pure chitosan, indicate interactions between hydroxyl groups of chitosan and magnetite. Additionally, the band at approximately 895 cm^−1^, attributed to saccharide ring vibrations, confirms that the chitosan backbone remained structurally intact after nanocomposite formation. The band observed in the 1558–1589 cm^−1^ region is assigned to the NH_2_ δ(NH_2_) deformation vibration. Its shift by δ = 16–31 cm^−1^ (to approximately 1558, 1573, and 1566 cm^−1^ for MCN-10, MCN-20, and MCN-30, respectively) indicates that interaction with magnetite occurs via the –NH_2_ groups.

Overall, the FTIR results demonstrate that magnetite nanoparticles were successfully bonded to chitosan without disrupting the biopolymer backbone. The systematic shifts in the amide bands with increasing magnetite content confirm the formation of strong interfacial interactions between chitosan functional groups and magnetite nanoparticles, indicating effective nanocomposite formation rather than simple physical mixing.

#### 3.1.4. Mössbauer Spectroscopy

**Figure 4:**
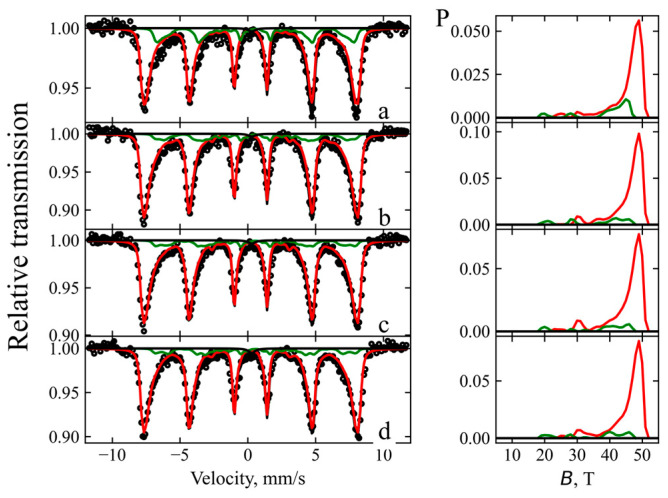
Mössbauer spectra of (a) synthesized Fe_3_O_4_ nanoparticles and magnetite–chitosan nanocomposites (b) MCN-30, (c) MCN-20, and (d) MCN-10, with the corresponding hyperfine field distributions shown on the right. Red and green lines represent the two hyperfine distributions, while the black line indicate their sum.

Mössbauer spectra of pure synthesized magnetite and chitosan-coated magnetite nanoparticles (Figure 4) were analyzed using two hyperfine field distributions and a singlet using the WinNormosDist software (Version: 3), exhibiting different isomer shifts (Table 1). These shifts are characteristic of tetrahedral A (Fe^3+^) and octahedral B (Fe^3+^/Fe^2+^) sites in the magnetite crystal structure. The spectral area ratio of the two hyperfine field distributions indicates that the relative amount of Fe^2+^, defined as *I*_Fe2+_ *=* 0.5*I_B_*/(*I_A_ + I_B_*) = 0.05–0.09, is characteristic of nonstoichiometric magnetite and also can be described as mixture of magnetite and maghemite resulting from the permanent atmospheric oxidation of Fe^2+^ during the degradation of the parent magnetite. The average size of magnetite nanoparticles was estimated to the approximately of 18 ± 1 nm using Equation (13) [45] and is in good correlation with calculated using XRD patterns.

(13) $$\langle B\rangle =B_{0}\left(1-\frac{kT}{2KV}\right)$$

This estimation takes into account a decrease of approximately 7% in the average hyperfine field *<B>* of both hyperfine field distributions compared to bulk magnetite values ($B_{0}^{A}\approx 49T;B_{0}^{B}\approx 46T$) [46], which is attributed to the reduced particle volume *V*. *K* is the magnetic anisotropy constant of the magnetite and *k* is the Boltzmann constant. An additional decrease in the relative area of the second sub-spectrum (sublattice B) in the magnetite–chitosan composites indicates further oxidation of the nanoparticles.

#### 3.1.5. Point of Zero Charge and Magnetization

**Figure 5:**
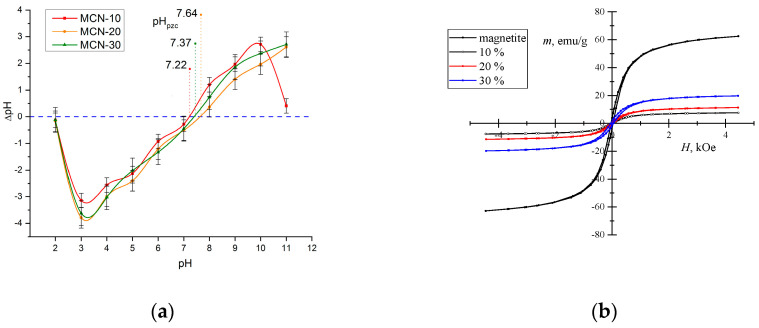
Effects of solution pH on MCN’s charge (**a**) and comparison of magnetization curves of synthesized Fe_3_O_4_ nanoparticles, MCN-10, MCN-20 and MCN-30 (**b**).

The data are plotted (Figure 5a), where pH refers to the initial value and Δ*pH* to the calculated (Equation (1)) difference. The point of zero charge (*pH_pzc_*) of the tested adsorbent corresponds to the pH at which Δ*pH* = 0. The determined *pH_pzc_* values for MCN-10, MCN-20, and MCN-30 were 7.22 ± 0.14, 7.64 ± 0.21, and 7.37 ± 0.18, respectively. This means that at pH values of used media higher than *pH_pzc_*, MCN’s surface will be negatively charged and favorable for positively charged adsorbates, while at pH values below *pH_pzc_*, it will be the opposite, where the MCN will be positively charged and favorable for interactions with negatively charged adsorbates [1].

The magnetization value (*m*, emu/g) was measured for the synthesized magnetite (62.7 emu/g) and for each MCN sample as a function of the strength of the magnetic field (*H*, kOe) (Figure 5b). For the synthesized magnetite–chitosan nanocomposites, the values were lower: 19.8 emu/g, 11.3 emu/g and 8.2 emu/g, corresponding to the Fe_3_O_4_ content, with MCN-30 > MCN-20 > MCN-10. This was further confirmed during adsorption experiments, where MCN-30 was separated after 25–40 s of exposure to magnetic force using a neodymium magnet (class N42), MCN-20 within 35–55 s, and MCN-10 within 40–90 s.

### 3.2. Adsorption Studies

#### 3.2.1. Removal Efficiency and Adsorption Capacity

**Figure 6:**
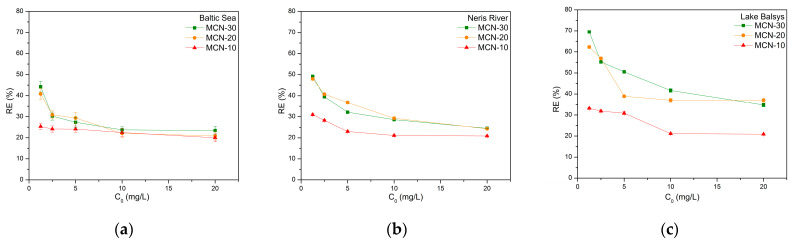
Dependence of removal efficiency (*RE*) on initial concentration (*C*_0_) of Co (II) using MCN-10, MCN-20, and MCN-30 in Baltic Sea (**a**), Neris River (**b**) and Lake Balsys (**c**) water samples.

The removal efficiency (*RE*) of MCN nanocomposites for Co (II) ions from the BS, NR, and LB water samples was calculated using Equation (2), and the results were plotted as a function of initial concentration *C*_0_ vs. *RE* (Figure 6).

The highest removal efficiency was observed in the Lake Balsys samples, with 69.5% for MCN-30, 62.3% for MCN-20, and 33.2% for MCN-10 at initial Co (II) concentrations of 1.25 mg/L, due to the lentic origin of the water samples, which allows for a less disrupted adsorption process. A similar trend was observed for the Baltic Sea and Neris River water samples (MCN-30 > MCN-20 > MCN-10), but with lower removal efficiencies, ranging from 49.2% to 25.4%. The river water samples typically exhibited a more complex aqueous matrix compared to the lake water, due to higher concentrations of suspended solids and dissolved organic matter, which can cause site competition, thereby reducing the adsorption efficiency. The lowest removal efficiency of MCNs for Co (II) in seawater samples can be attributed to the high ionic strength and salinity, which results in the shielding of the adsorbent’s active sites. It is important to note that a more detailed characterization of water composition, including the dissolved organic matter, ionic composition, hardness, and competing ions, was not conducted in this study, but has been described in our previous publications [47,48]. These factors are known to influence adsorption through competitive binding, complexation, and changes in metal speciation [49,50,51]. Overall, MCN-20 and MCN-30 exhibited higher removal efficiency than MCN-10. These results indicate that MCN-30 is the most effective for the adsorption of Co (II) ions in different aquatic environments, while also providing the best magnetic separation properties.

**Figure 7:**
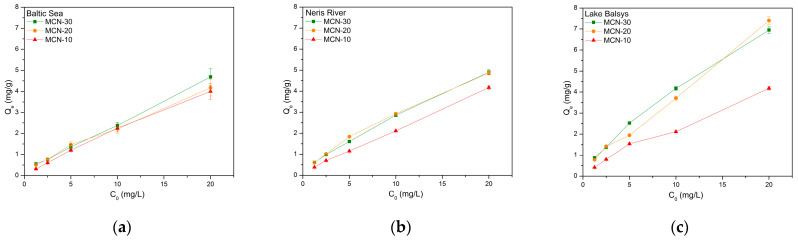
Dependence of adsorption capacity (*Q_e_*) on initial concentration (*C*_0_) of Co (II) using MCN-10, MCN-20, and MCN-30 in Baltic Sea (**a**), Neris River (**b**) and Lake Balsys (**c**) water samples.

The achieved adsorption capacity (*Q_e_*, Equation (3)) from the batch sorption experiments is shown in Figure 7, where the data are plotted as Q_e_ versus the initial concentration C_0_ of Co (II) ions. The highest adsorption capacities were measured in the LB samples, with 7.39 mg/g using MCN-20, and in the NR and BS samples using MCN-30, with 4.9 mg/g and 4.7 mg/g, respectively, at the highest initial concentration used, 20 mg/L. All these data were used for the isothermal studies presented below.

The results demonstrate acceptable Co (II) adsorption levels in all tested media. Adsorption levels increased with a higher percentage of magnetite in the sorbent as follows: 10 < 20 < 30 wt. % magnetite. This may indicate that magnetite nanoparticles attached to the surface of chitosan were better positioned for cobalt adsorption in this nanocomposite and interacted positively in the media used. It is also notable that the salinity of the media affected adsorption efficiency, with MCN-30 achieving a maximum of 46% in Baltic Sea water, compared to fresh water samples with about 49% in Neris River and 69% in Lake Balsys. This difference can be attributed to variations in water chemistry between marine and freshwater environments. Seawater is characterized by significantly higher ionic strength and salinity, containing high concentrations of competing cations such as Na^+^, Mg^2+^, and Ca^2+^. These ions may compete with Co^2+^ for adsorption sites on the amino and hydroxyl functional groups of chitosan and partially shield electrostatic interactions between the sorbent surface and cobalt ions. In contrast, freshwater systems generally contain lower concentrations of competing ions, which facilitates stronger interaction between Co^2+^ and the active adsorption sites of the MCN. A similar trend was observed for MCNCS nanocomposite in [52] using laboratory samples of Co^2+^, where the highest removal efficiency occurred at pH = 8 within a pH range from 5 to 8, achieving adsorption capacity of 17.92 mg/g. M. Sharifi et al. reported a higher removal efficiency of about 90% at pH = 5.5 and about 70% at pH = 7.5 for a similar CMNC nanocomposite, using a simple laboratory-made Co (II) solution [42].

#### 3.2.2. Adsorption Isotherm Modeling

The sorption capacities of MCN-10, MCN-20, and MCN-30 for Co (II), traced with ^60^Co radionuclide and obtained from sorption experiments in BS, NR, and LB water samples, were analyzed using adsorption isotherms: Freundlich, Langmuir, Temkin, and Harkins–Jura. The parameters for all models (Table 2) were calculated from the equilibrium data, and the linear forms (Equations (4)–(8), respectively) of the isotherms were fitted for comparison.

Linearized forms of adsorption isotherm models were used for comparative purposes; however, it is recognized that such approaches may introduce bias in parameter estimation and that isotherm models are primarily empirical representations rather than direct indicators of adsorption mechanisms [53,54]. Therefore, the interpretation of adsorption behavior was made with caution, considering both model fitting and physicochemical characteristics of the adsorbent.

**Figure 8:**
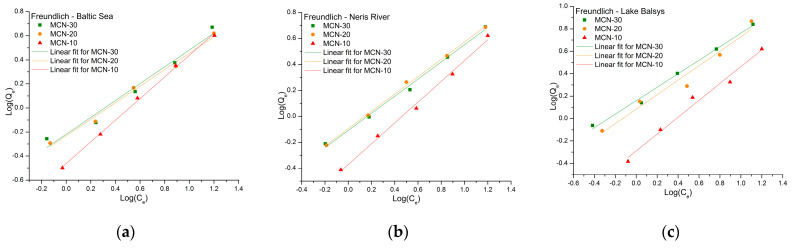
Freundlich isotherm linear fit for the adsorption of Co (II) on MCN-10, MCN-20 and MCN-30 in water samples from the Baltic Sea (**a**), Neris River (**b**) and Lake Balsys (**c**).

Figure 8 presents the results of the linear fit of the Freundlich isotherm for each sorbent in the specified water sample. The calculated correlation coefficients (R^2^) ranged from 0.951 (for MCN-20 in LB) to 0.998 (for MCN-10 in BS), the highest among the isotherms studied. This indicates that the Freundlich isotherm provides the best empirical fit among the tested models. The adsorption intensity factors (1/n values) ranged from 0.602 to 0.899 in all samples, meeting the condition 0 < 1/n < 1, which indicates that adsorption is favorable. A higher K_F_ indicates better adsorption efficiency; the highest value was observed for MCN-30 in LB, confirming the best removal efficiency for Co (II) in this study.

**Figure 9:**
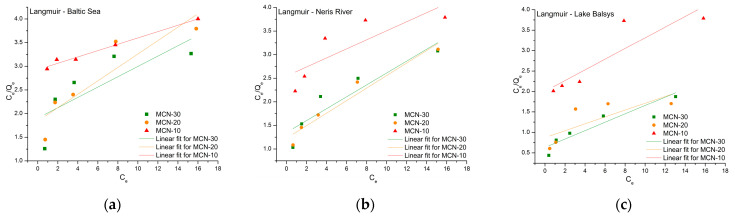
Langmuir isotherm linear fit for the adsorption of Co (II) on MCN-10, MCN-20 and MCN-30 in water samples from the Baltic Sea (**a**), Neris River (**b**) and Lake Balsys (**c**).

The Langmuir isotherm fit shown in Figure 9 for MCNs produced less correlated results, with *R*^2^ values ranging from 0.462 (for MCN-20 in Lake Balsys) to 0.979 (for MCN-10 in the Baltic Sea). The highest Langmuir coefficient, *K_L_* (0.162), was observed for MCN-30 in Balsys Lake. All calculated *R_L_* values (Equation (6)) ranged from 0.236 to 0.686, meeting the criteria that 0 < *R_L_* < 1 [55], which means the reaction was favorable in all studied cases. The maximum adsorption capacity (*Q_max_*) according to the Langmuir isotherm fit was highest for the MCN-10 nanocomposite in Baltic Sea water, at 14.9 mg/g, indicating that chitosan functional groups participate more actively in heavy metal and radionuclide adsorption under higher salinity conditions. Other *Q_max_* values were not lower than 7.06 mg/g, with an average of 9.59 mg/g, indicating overall good adsorption performance in natural water samples.

**Figure 10:**
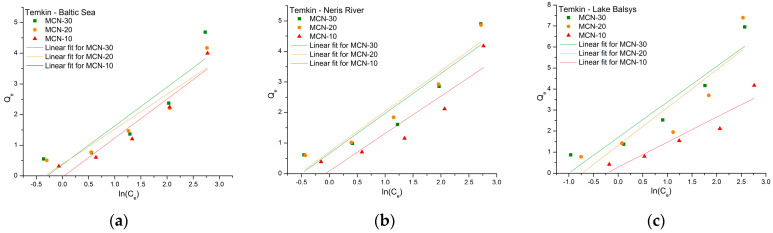
Temkin isotherm linear fit for the adsorption of Co (II) on MCN-10, MCN-20, and MCN-30 in water samples from the Baltic Sea (**a**), Neris River (**b**) and Lake Balsys (**c**).

The Temkin isotherm fit correlation coefficients were lower than those for the Freundlich isotherm, but in some cases higher than those for the Langmuir isotherm, ranging from 0.730 to 0.901 (for MCN-20 in LB and NR, respectively) (Figure 10). The calculated *K_T_* values, which indicate the interactions between adsorbates and adsorbent, were higher than 1, except for MCN-10, with values of 0.971, 1.07, and 1.27, which can be interpreted as being close to unity. This indicates that for MCN-20 and MCN-30, adsorption is favorable and efficient, while for MCN-10, it is moderate or weak. Values lower than 1, which would indicate an unfavorable adsorption process, were not observed. The *B* parameters ranged from 1.14 to 1.80, which are higher than 1 and indicate strong bonding and interaction between adsorbate and adsorbent—in this case, between MCNs and Co (II).

**Figure 11:**
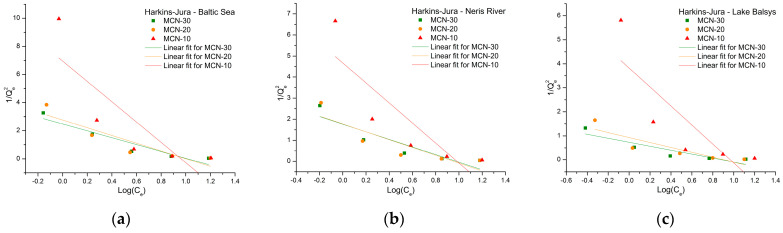
Harkins–Jura isotherm linear fit for the adsorption of Co (II) on MCN-10, -20 and -30 in water samples from the Baltic Sea (**a**), Neris River (**b**) and Lake Balsys (**c**).

The final isotherm used to investigate adsorption parameters was the Harkins–Jura model (Figure 11), which is commonly applied to describe multilayer (usually liquid) adsorption of adsorbate onto the heterogeneous surface of the adsorbent and enables calculation of the active relative surface area of the adsorbent. The highest *A_HJ_* constant, 1.20, was calculated for MCN-30 in LB, indicating a relatively higher surface area of MCN-30 for possible interaction with the adsorbate; for the others, the values were lower (*A_HJ_* < 1), indicating a lower viable area for Co (II) adsorption. Another constant, *B_HJ_*, describes the adsorption capacity and interaction factor in a similar way (*B_HJ_* > 1 is preferable). The calculated *B_HJ_* values in our case were around 1.1 ± 0.1, except for MCN-20 in LB, which had a value of 1.13. The correlation coefficients ranged from 0.591 to 0.865.

The low correlation for the Harkins–Jura model (0.592 < *R*^2^ < 0.865), compared to those of the Freundlich and Langmuir models, suggests that Co (II) adsorption does not occur through the formation of a multilayer, liquid film-like covering.

In summary, the adsorption process occurs on a heterogeneous surface and involves a combination of interactions. While the Freundlich model indicates surface heterogeneity and suggests the possibility of multilayer adsorption, it should not be interpreted as definitive evidence of a multilayer mechanism. The agreement with the Langmuir and Temkin models suggests that site-specific interactions also contribute to the adsorption process. Therefore, the process can be described as involving contributions from both chemisorption and weaker physical interactions rather than a single dominant mechanism. The observed adsorption behavior can be attributed to the interactions between Co (II) ions and the functional groups (-NH_2_, -OH) present within the chitosan matrix. This finding aligns with the conclusions reported by other researchers [52,55]. A similar behavioral pattern has been documented in the context of magnetic chitosan-based nanocomposite sorbents. In such instances, the adsorption process is typically characterized as occurring on heterogeneous surfaces, with contributions from both specific binding and weaker interactions [54,56,57]. In addition, the same trends have been reported while using adsorbents similar [42] or comparable chitosan-based nanocomposites [55,58] for the adsorption of heavy metal ions (Ni (II); Cu (II), Cd (II), Zn (II); Cu (II), Cd (II), respectfully), with better values of correlation coefficient *R*^2^ ranging from 0.903 for Temkin, from 0.9266 for Langmuir and from 0.913 for Freundlich. These values are likely associated with the use of synthetic media, which provide favorable conditions. In the present study, slightly lower *R*^2^ values were observed, which may be attributed to the increased chemical complexity of natural water matrices. Furthermore, the *Q_max_* values obtained in this study in seawater (14.9 mg/g) and in freshwater (9.59 mg/g) compared to other studies were similar (17.41 mg/g in synthetic media [52]) or lower (53.49 mg/g in synthetic media [42]), indicating greater chemical complexity in real conditions. It should be noted that adsorption experiments in this study were performed at the natural pH of the water samples and at a constant temperature to reflect environmental conditions; therefore, the proposed adsorption mechanism should be considered as indicative rather than definitive.

#### 3.2.3. Adsorption Kinetics Modeling

**Figure 12:**
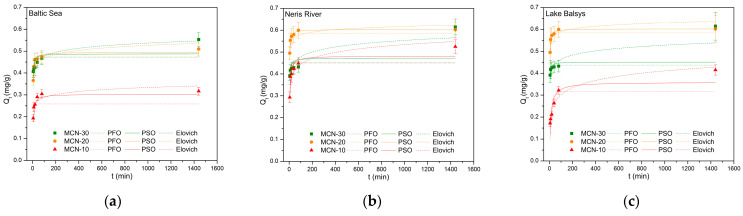
Plots of *Q_t_* against *t* and fitted PFO, PSO, and Elovich kinetic models of Co (II) adsorption on MCN-10, MCN-20, and MCN-30 in Baltic Sea (**a**), Neris River (**b**) and Lake Balsys (**c**) water samples.

The sorption kinetics were studied using the same dosage of adsorbent and target ions and varying only the contact time. The results showing the dependence on contact time (*t* vs. *Q_t_*) were plotted (Figure 12) and analyzed using the OriginPro program by non-linear fitting of the pseudo-first-order (PFO), pseudo-second-order (PSO), and Elovich kinetic model (Equations (10), (11), (12), respectively). The calculated constants for all models are presented in Table 3.

The observed correlation coefficients (*R*^2^) were higher for the PSO model (MCN-10, MCN-20 in BS; MCN-10, MCN-20 in NR; and MCN-20 in LB) and, in some cases, for the Elovich model (MCN-30 in BS, MCN-30 in NR, MCN-10, MCN-30 in LB) than for the PFO kinetic model. The calculated k_2_ rate constants of PSO increased in the sequence MCN-10 < MCN-20, indicating that MCN-20 achieved a faster adsorption kinetics for Co (II) in each of the tested media, with values of 1.157 g/(mg min) in BS, 1.574 g/(mg min) in NR, and 1.580 g/(mg min) in LB. Moreover, the Q_e_ values for MCN-20 exhibited slightly better results compared to others and were in the same range from 0.305 to 0.603 mg/g, indicating a similar thermodynamic affinity for all MCNs and supporting the conclusion that a stable chemisorption mechanism governs the process for all three adsorbents. The Elovich kinetic model provided the best fit for the MCN-30 nanocomposite adsorption data across all tested media. While the PSO model assumes a more uniform surface, in our case, MCN-10 and MCN-20 had a higher number of -NH_2_ and -OH active groups on their chitosan surfaces compared to MCN-30. However, the Elovich model accounts for the energetic heterogeneity introduced during the modification process, which aligns with the fact that MCN-30 was decorated with highest amount of Fe_3_O_4_ nanoparticles. The high α values for MCN-30 (4.64 g/(g min) in LB and 23.341 g/(g min) in BS) reflects a rapid initial uptake, while the linear relationship between *Q_t_* and *ln*(*t*) confirms that the adsorption rate decreased exponentially as the heterogeneous surface sites were progressively occupied. In addition, this indicates that both the persistence of active groups (-NH_2_ and -OH) and magnetite nanoparticles on the chitosan surface actively participated in the adsorption process, providing more efficient adsorption of Co (II). The lowest chi-square (χ^2^) values, in cases where R^2^ values are similar between the PSO and Elovich models, indicate the best fit of the given model and that the coefficients were calculated in the most accurate and relevant way. These results confirm that the interaction between adsorbent (MCNs) and adsorbate (Co (II)) occurs via chemisorption, providing higher adoption efficiency when increasing the heterogeneity of the surface in the following sequence: MCN-10 < MCN-20 < MCN-30.

These results are consistent with previous findings in the study by [58], which reported similar kinetic behavior for different adsorbates (Cu (II), Cd (II)) in synthetic media using a similar adsorbent. Compared to [42,52,55], which utilized comparable chitosan-based adsorbents, the observed trends were similar, demonstrating consistency with both the PSO and Elovich kinetic models. The R^2^ values obtained in this study were slightly lower than those reported in the literature because reduced correlation is attributed to the use of natural aqueous matrices, which introduce greater chemical complexity compared to the synthetic solutions or deionized water used by other researchers.

A direct quantitative comparison with the literature data is complicated by differences in experimental conditions, as many studies are conducted in simplified synthetic systems. In contrast, the present study evaluated adsorption performance in natural water matrices with higher chemical complexity, which may affect both adsorption capacity and kinetic parameters. However, the obtained adsorption capacities and kinetic parameters are comparable to those reported for analogous magnetic chitosan-based sorbents under comparable experimental conditions, taking into account the differences between synthetic and natural water matrices [42,46,55]. While the potential for regeneration and reuse was not examined in this study, the magnetic properties of the nanocomposites suggest that efficient recovery is possible using an external magnetic field. Previous studies have demonstrated that magnetic chitosan-based nanocomposite sorbents can be regenerated and reused over multiple adsorption–desorption cycles with acceptable performance [59,60]. While the investigation did not encompass the examination of long-term stability and leaching behavior, it is hypothesized that the implementation of glutaraldehyde crosslinking will augment the structural stability of the chitosan matrix and mitigate its solubility. This assertion is substantiated by the documented findings in crosslinked chitosan systems [61,62]. However, it is important to note that chitosan-based materials may still undergo swelling or partial degradation under environmental conditions. Furthermore, previous studies on magnetic chitosan-based materials have demonstrated stable performance in aqueous systems during repeated use [59]. Consequently, further studies are necessary to assess the long-term stability, potential leaching, and environmental safety of the nanocomposites in natural water systems.

## 4. Conclusions

Magnetite–chitosan nanocomposites (MCNs) with controlled Fe_3_O_4_ loadings of 10, 20, and 30 wt. % were successfully synthesized via a crosslinking method, demonstrating reproducible structural and magnetic properties confirmed by characterization. Experimental results revealed that the optimum magnetization of 11.3 emu/g was observed for MCN-20, allowing easy and rapid separation of magnetic nanocomposites using a magnetic field under 1 min. This suggests that higher (≥20 wt. %) loads of magnetite ensure effective magnetic properties of the MCNs. The highest removal efficiencies observed using MCN-30 were 44.3%, in Baltic Sea water, about 49.2% in Neris River water, and about 69.5% in Lake Balsys water, suggesting that even higher values could be achieved at Co (II) concentrations below 1.25 mg/L. The highest adsorption capacity was registered in Lake Balsys water samples, at 7.39 mg/g using MCN-20; in Neris River and Baltic Sea water, the values were lower, at 4.9 mg/g and 4.7 mg/g, respectively, using MCN-30. The maximum adsorption capacities of MCNs for Co (II), calculated using the Langmuir isotherm model, ranged from 7.05 to 14.93 mg/g. Adsorption isotherm studies showed that the equilibrium data best fit the Freundlich model, indicating favorable multilayer adsorption of Co (II) on the heterogeneous surface of MCNs. The kinetic behavior of the adsorption process followed the PSO and Elovich models, confirming chemisorption processes on the heterogeneous surface of MCN and showing that equilibrium was reached within 120 min. Notably, these results can be considered for ^60^Co radionuclide, which was used as a tracer, and may expand the applicability of MCN in radioactive waste management or as a pre-concentrator for cobalt-60 in aquatic media. Although MCN is a modified biodegradable polymer and its composition should not have any negative impact on the environment, it is preferable to confirm the eco-friendliness and stability of the adsorbent through ecotoxicological or long-term exposure studies on aquatic organisms before use.

## Figures and Tables

**Table 1:** Parameters of Mössbauer spectrum: I is the relative area of hyperfine field distribution corresponding to the A and B sublattice of magnetite; δ is the isomer shift relative to α-Fe; <B> is the average hyperfine field.

Sample	** *I*, %	*δ*, mm/s	<*B*>, T	
Magnetite	81	0.32 ± 0.01	45.6	Mag. A (Fe^3+^)
(Fe_3_O_4_ 100 wt. %)	18	0.67 *	39.5	Mag. B (Fe^2+^ + Fe^3+^)
	1	0.4 *	-	
MCN-10	89	0.33 ± 0.01	45.7	Mag. A (Fe^3+^)
(Fe_3_O_4_ 10 wt. %%)	10	0.67 *	36.3	Mag. B (Fe^2+^ + Fe^3+^)
	1	0.4 *	-	
MCN-20	88	0.32 ± 0.01	45.4	Mag. A (Fe^3+^)
(Fe_3_O_4_ 20 wt. %)	11	0.65 *	37.6	Mag. B (Fe^2+^ + Fe^3+^)
	1	0.4 *	-	
MCN-30	87	0.32 ± 0.01	45.9	Mag. A (Fe^3+^)
(Fe_3_O_4_ 30 wt. %)	12	0.65 *	36.2	Mag. B (Fe^2+^ + Fe^3+^)
	1	0.4 *	-	

* fixed, ** I (%) represents the relative spectral area of the fitted Mössbauer components and does not directly correspond to the mass or molar fraction of Fe in the samples.

**Table 2:** Parameters of isotherm models derived from linear fits for Co (II) adsorption on MCN-10, MCN -20, and MCN -30 in water samples from the Baltic Sea, Neris River and Lake Balsys.

Isotherm Model		Baltic Sea			Neris River			Lake Balsys		
		MCN-10	MCN-20	MCN-30	MCN-10	MCN-20	MCN-30	MCN-10	MCN-20	MCN-30
Freundlich	1/*n*	0.900	0.687	0.701	0.801	0.667	0.660	0.752	0.646	0.602
	*K_F_*	0.630	0.793	0.803	0.690	0.910	0.897	0.749	1.088	1.173
	*R* ^2^	0.998	0.986	0.963	0.993	0.998	0.993	0.977	0.951	0.991
Langmuir	*Q_max_*	14.934	7.056	9.157	10.424	7.524	7.935	7.659	11.916	9.768
	*K_L_*	0.023	0.077	0.057	0.038	0.108	0.093	0.065	0.096	0.162
	*R_L_*	0.686	0.394	0.466	0.570	0.316	0.349	0.435	0.342	0.236
	*R* ^2^	0.979	0.754	0.500	0.577	0.930	0.812	0.739	0.462	0.887
Temkin	*K_T_*	0.971	1.407	1.338	1.076	1.717	1.653	1.266	2.079	2.732
	*B*	1.272	1.138	1.265	1.223	1.324	1.310	1.190	1.799	1.688
	*R* ^2^	0.882	0.834	0.768	0.809	0.902	0.847	0.856	0.730	0.863
Harkins-Jura	*A_HJ_*	0.138	0.363	0.409	0.210	0.537	0.552	0.253	0.968	1.202
	*B_HJ_*	1.040	0.998	0.986	1.018	1.048	1.025	1.034	1.108	1.133
	*R* ^2^	0.612	0.774	0.865	0.670	0.696	0.771	0.592	0.713	0.779

**Table 3:** The parameters of kinetic models (PFO, PSO, and Elovich) calculated by analysis of determined results for dependence of contact time for Co (II) adsorption on MCN-10, MCN-20, and MCN-30 in Baltic Sea, Neris River, and Lake Balsys water samples (C_0_ = 1.25 mg/L, adsorbent dose = 1 g/L, contact time = 5–1440 min, T = 15 °C).

Kinetic Model		Baltic Sea			Neris River			Lake Balsys		
		MCN-10	MCN-20	MCN-30	MCN-10	MCN-20	MCN-30	MCN-10	MCN-20	MCN-30
PFO	*Q_e_*	0.259	0.474	0.470	0.452	0.585	0.450	0.316	0.584	0.437
	*k*_1_	−0.248	−0.290	−0.385	−0.202	−0.363	−0.390	−0.762	−0.366	−0.442
	*R* ^2^	0.305	0.883	0.158	0.741	0.863	−0.015	0.399	0.820	−0.037
	*χ* ^2^	5.224	0.459	2.548	1.994	0.208	5.916	13.377	0.124	2.051
PSO	*Q_e_*	0.303	0.495	0.488	0.482	0.603	0.482	0.360	0.602	0.451
	*k*_2_	0.917	1.157	1.676	0.632	1.574	1.713	0.277	1.580	2.526
	*R* ^2^	0.861	0.963	0.435	0.882	0.978	0.163	0.727	0.967	0.089
	*χ* ^2^	1.048	0.147	1.710	0.905	0.034	4.878	6.091	0.023	1.803
Elovich	*α*	4.1 × 10^2^	2.9 × 10^4^	2.3 × 10^4^	3.4 × 10^1^	3.1 × 10^11^	6.3 × 10^2^	2.9 × 10^−1^	4.4 × 10^10^	4.6 × 10^3^
	*β*	50.505	39.571	38.371	25.601	60.691	30.294	21.216	56.204	35.855
	*R* ^2^	0.692	0.724	0.979	0.867	0.549	0.766	0.943	0.516	0.644
	*χ* ^2^	2.315	1.088	0.062	1.026	0.687	1.366	1.261	0.333	0.705

## Data Availability

The original contributions presented in this study are included in the article/Supplementary Materials. Further inquiries can be directed to the corresponding author.

## Supplementary Materials

The following supporting information can be downloaded at https://www.mdpi.com/article/10.3390/nano16070393/s1: Figure S1. SEM image of MCN-30 (a) and size distribution (b) of the nanocomposite.

## Abbreviations

The following abbreviations are used in this manuscript:

MCN(s)	Magnetite–chitosan nanocomposite(s)
MCN-10	Magnetite–chitosan nanocomposite loaded with Fe3O4 10 wt. %
MCN-20	Magnetite–chitosan nanocomposite loaded with Fe3O4 20 wt. %
MCN-30	Magnetite–chitosan nanocomposite loaded with Fe3O4 30 wt. %
BS	Baltic Sea water sample
NR	Neris River water sample
LB	Lake Balsys water sample
TEM	Transmission electron microscopy
SEM	Scanning electron microscopy
XRD	X-ray diffraction
FTIR	Fourier transform infrared spectroscopy
PFO	Pseudo-first-order kinetic model
PSO	Pseudo-second-order kinetic model
MCNCS	Magnetic cyanoethyl chitosan beads [52]
CMNC	Chitosan—magnetite nanocomposite [42]
