# Supplementary material for: Different Magnetization Levels of Magnetite–Chitosan Nanocomposites for Co (II) Adsorption from Natural Waters
Source: Nanomaterials (Basel). 2026 Mar 25;16(7):393. doi: 10.3390/nano16070393 (PMC13074300; doi:10.3390/nano16070393)
Supplement: Supplementary file 1 [file nanomaterials-16-00393-s001.zip › nanomaterials-4212352-supplementary.pdf]

Supplementary File

Different Magnetization Levels of Magnetite–Chitosan Nano-Composites for Co (II) Adsorption from Natural Waters

Sergej Šemčuk[1,2,*], Živilė Jurgelėnė[2], Vidas Pakštas[1], Danguolė Montvydienė[2], Audrius Drabavičius[1], Kęstutis Jokšas[2], Martynas Talaikis[1], Jonas Mažeika[2], Kęstutis Mažeika[1], Karina Kuzborskaja[1], Galina Lujanienė[1]

[1]State Research Institute Center for Physical Sciences and Technology (FTMC), Savanorių ave. 231, 02300 Vilnius, Lithuania

[2]State Scientific Research Institute Nature Research Centre (NRC), Akademijos St. 2, 08412 Vilnius, Lithuania

*Correspondence: sergej.semcuk@ftmc.lt

The surface morphology and size distribution of MCN-30 was archeved using scanning electron microscopy (SEM) on Helios NanoLab 650 (FEI, Netherlands, 2011).

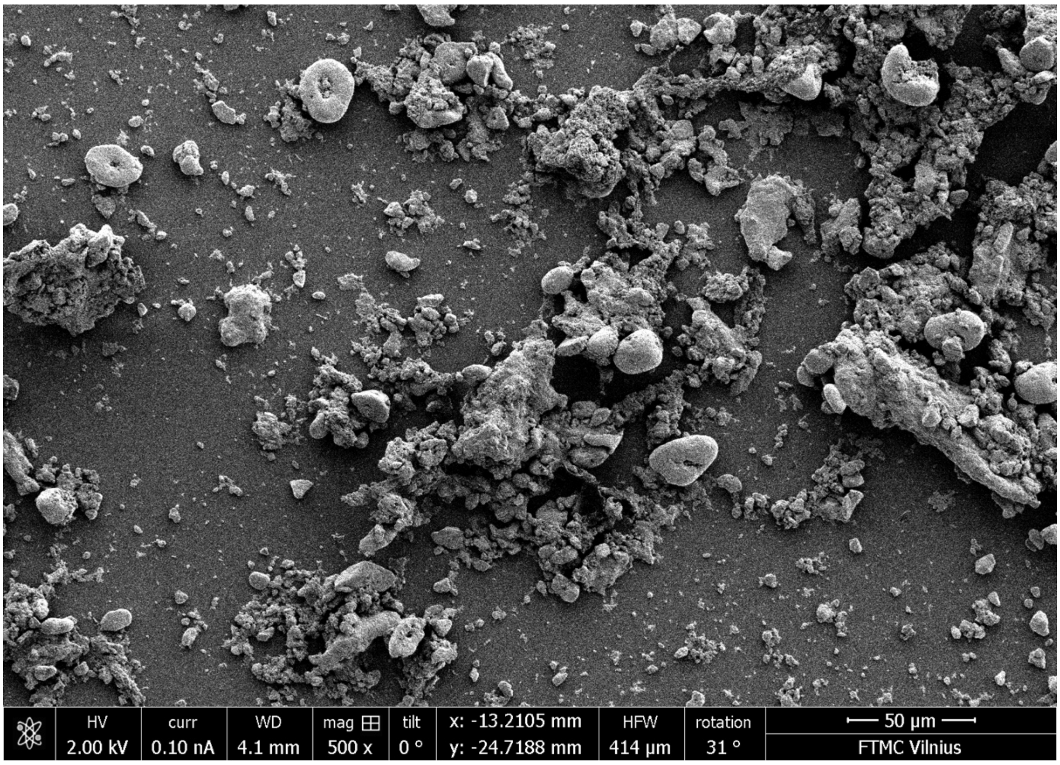

(a)

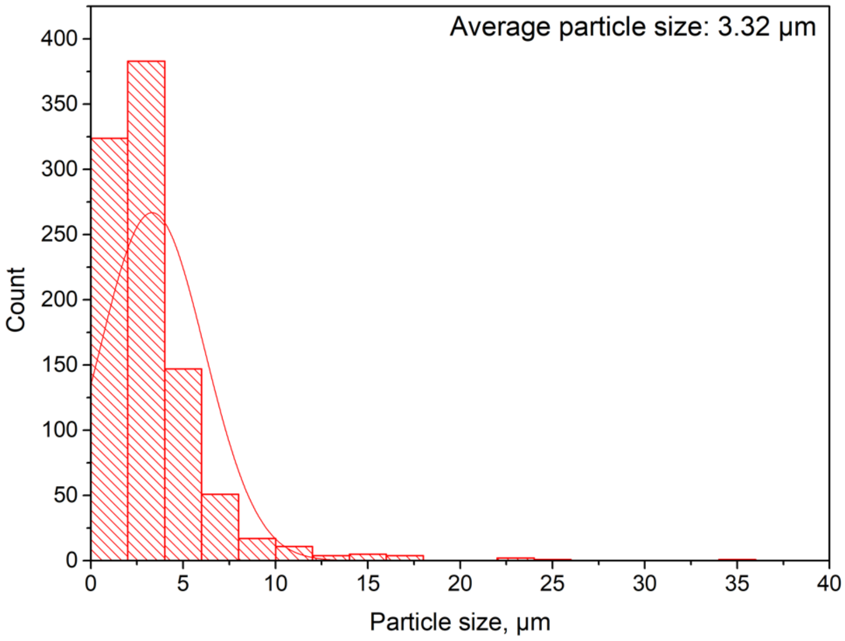

(b)

Supplementary Figure S1. SEM image of MCN-30 (a) and size distribution (b) of the nanocomposite.
